# Gastric Schwannoma in an Octogenarian: A Case Report and Review of the Literature

**DOI:** 10.7759/cureus.58857

**Published:** 2024-04-23

**Authors:** Verena Sorial, Aramish S Khan, Terry Welsh, Lei Zhang, Sameh A Fayek

**Affiliations:** 1 Surgery, University of the Incarnate Word School of Osteopathic Medicine, San Antonio, USA; 2 Pathology, Parkview Community Hospital Medical Center, Riverside, USA; 3 Surgery, University of California Riverside, Riverside, USA; 4 Surgery, Faculty of Medicine, Cairo, EGY

**Keywords:** laparoscopic resection, antoni structures, gastrointestinal stromal tumor (gist), neurofibromatosis ii, s100 protein, octogenarian, gastric schwannoma

## Abstract

Gastric schwannomas are an exceedingly rare tumor arising from the myenteric plexus of the gastrointestinal enteric nervous system. These schwannomas are most commonly benign and reported to occur in female patients with a mean age of 58 at presentation. They are most often discovered incidentally, but can occasionally present with abdominal discomfort, obstructive symptoms, or GI bleeding. Frequently, the initial clinical consideration is for a gastrointestinal stromal tumor, which is much more common. A definitive diagnosis is made with microscopic imaging and immunohistochemical staining. Complete surgical resection, typically performed laparoscopically, is the most definitive and usually curative treatment, requiring no further follow-up. Herein, we present the first and only case of gastric schwannoma in an octogenarian and discuss an update on current diagnostic and therapeutic options.

## Introduction

Schwannomas (also known as neuromas, neurinomas "of Verocay," and neurilemmomas) are well-encapsulated, slowly growing, usually benign, and are the most common nerve sheath tumors, caused by the excessive proliferation of the myelin-producing Schwann cells derived from the neural crest [1]. These tumors can originate from any myelinated central or peripheral nerves with Schwann cells. Schwannomas are solitary in 90% of the cases, with the majority (60%) consisting of vestibular schwannomas [1]. Other more commonly reported locations include the upper limbs, followed by the head, trunk, and flexor surfaces of the lower extremities. More rare locations include the posterior mediastinum, retroperitoneum, spinal roots, bone, gastrointestinal tract, pancreas, liver, thyroid, adrenal glands, and lymph nodes [2]. Multiple tumors in a patient should bring attention to syndromic associations (such as neurofibromatosis type 2, schwannomatosis, and Carney complex) [1]. 

Gastrointestinal schwannomas are exceedingly rare, with most reported cases in the stomach rather than the colon, rectum, esophagus, or small intestine. Gastric schwannomas represent 0.25% of all types of gastric tumors with the most common location being the stomach body (50%) followed by the antrum and fundus of the stomach [3]. While schwannomas in general are mostly solitary, gastric schwannomas are usually associated with other tumors and are part of neurofibromatosis 2 (NF2) [4]. Gastric schwannomas arise from the myenteric plexus within the muscularis propria layer, with spindle cells invading into the muscularis and submucosal layers; they are often incidentally discovered. In symptomatic cases, vague abdominal pain is the leading symptom. Other reported presentations include gastrointestinal obstruction, bleeding, or symptoms related to mass effects on nearby structures (liver, pancreas, or spleen) causing jaundice, pain, fatigue, or anemia. Imaging is often insufficient for a definitive diagnosis, however, microscopic examination and immunohistochemical stains clinch the diagnosis [3]. Of the reported cases, most patients with gastric schwannomas were females in the age range of 51-60 years old, with a mean age of 58 [5,6]. Herein, we present the first and only case of symptomatic isolated gastric schwannoma in an octogenarian man.

## Case presentation

**Figure 1:**
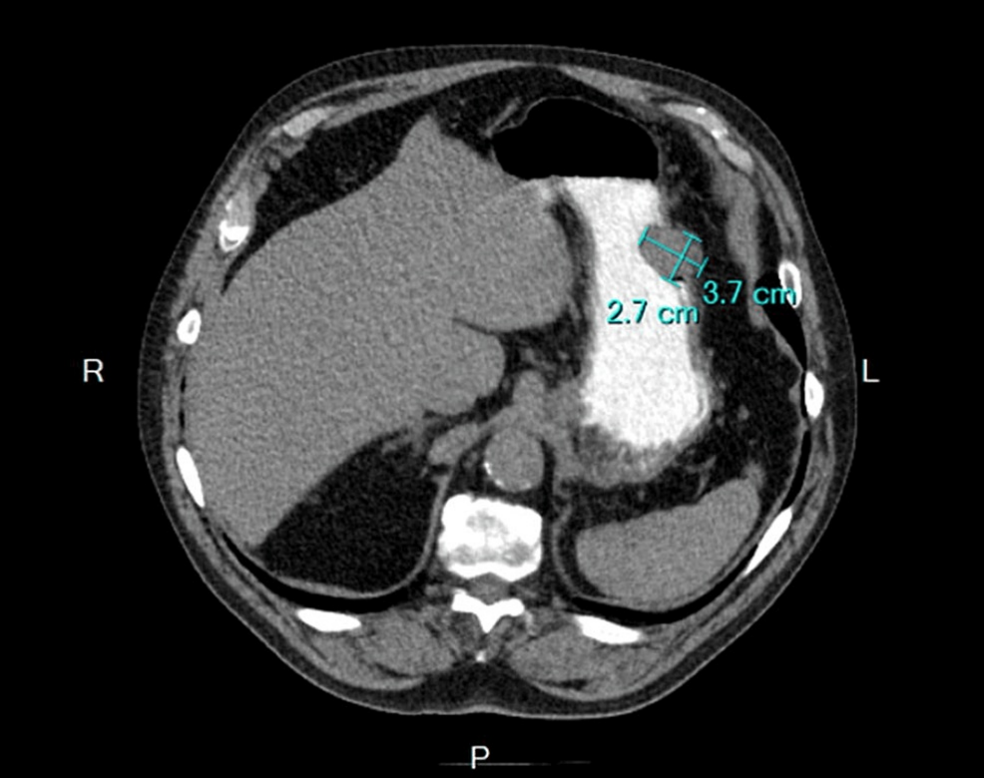
Pre-operative axial CT of the patient. Pre-operative axial CT of the patient showed a 3.7x2.7 cm fairly circumscribed intramural mass in the greater curvature of the stomach; no other abdominal masses were seen.

An 82-year-old patient, in a good state of health and otherwise stable, presented with abdominal pain to the emergency department. A computed tomography (CT) scan revealed a mass of approximately 4 cm at the greater curvature of the stomach, with initial consideration for a gastrointestinal stromal tumor (GIST) (Figure 1).

**Figure 2:**
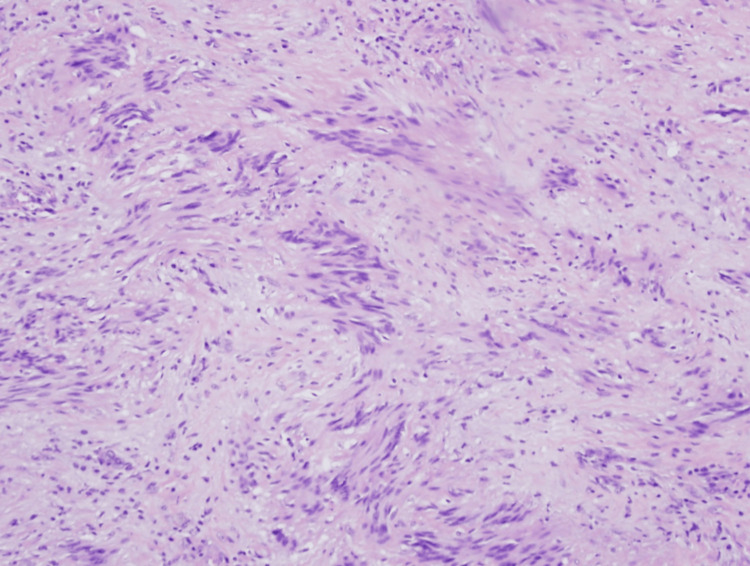
Histological features of the gastric mass. Histology demonstrating Antoni A and Antoni B regions suggestive of a schwannoma. Antoni A refers to the highly cellular regions seen in a stacking arrangement composed of spindle cells, with some curved and wavy hypercellular nuclei demonstrating nuclear palisading. Antoni B regions refer to the loose and hypocellular tissue, which exhibits a prominent extracellular matrix and secretion of laminin surrounding the Antoni A. These two findings together form Verocay bodies, the histologic characteristic feature of schwannomas.

**Figure 3:**
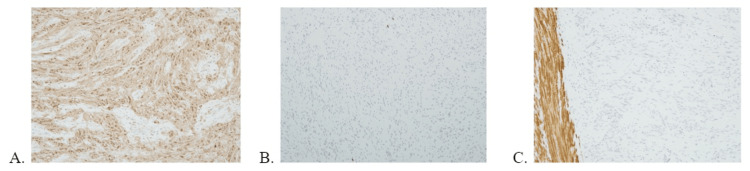
Immunohistochemical staining (A): positive S-100, indicative of schwannoma, (B): negative staining for C-KIT ruling out GIST, and (C): negative for SMA ruling out leiomyoma (the positive stain in normal muscularis propria, not the lesion).

The patient underwent a diagnostic laparoscopy where a gastric mass at the greater curvature was identified, and a stapled wedge gastric resection was performed. The patient had an uneventful postoperative course and was discharged home on postoperative day 4. Upon pathologic examination, it was found that the mass was present in the muscularis propria and extended into the submucosa; the mass was rimmed with lymphoid tissue. Histologically, the mass was formed by spindle cells and a few mitotic figures with Antoni A and Antoni B tissues, highly suggestive of schwannoma (Figure 2) [4,7]. Immunohistochemical staining demonstrated tumor cells to be positive for S100, and negative for CD34, CD117, DOG1, desmin, and smooth muscle actin (SMA) (Figure 3). The resected mass demonstrated a low Ki-67 index of 2-3%. Furthermore, the six resected lymph nodes were negative for malignancy.

## Discussion

Gastric schwannomas are exceedingly rare, and in previous cases have more commonly been seen in females, specifically ages 51-60. Although schwannomas are generally solitary, gastric schwannomas are usually associated with other tumors and are part of neurofibromatosis 2 (NF2) [4]. Most reported cases were found incidentally during the workup for another disease process [8]. This case report is unique with an isolated symptomatic gastric schwannoma in an octogenarian male that is not associated with NF2 or other genetic conditions. In rare cases where gastric schwannomas are symptomatic, the most common presentation is vague abdominal pain, as in the presented cases, that usually prompts a CT scan revealing a gastric mass. 

As for every single gastric schwannoma, there are about forty-five corresponding gastrointestinal stromal tumors (GIST) diagnosed; the finding of intramural gastric masses usually brings the clinical consideration of GIST rather than a schwannoma. Occasionally, CT scan findings could hint at the diagnosis where in the case of GISTs, the lesion often displays heterogeneous enhancement, central necrosis, and possible calcification with exophytic growth into the lumen. On the other hand, gastric schwannomas typically reveal well-defined margins, homogenous enhancement with a lack of necrosis, and less frequent calcifications [9, 10]. However, such findings may not be obvious and are not diagnostic. 

Further workup can include endoscopic ultrasound and fine needle aspiration, which are considered safe for sampling for pathological examination and confirmation of the nature of the disease [11]. The diagnostic accuracy is reported to be within the 43.3% to 52% range related usually to inadequate sampling resulting in this high rate of false negative results [2]. In addition, potential risks include tumor rupture and spread, which can negatively impact the outcomes. Therefore, the benefit vs. risk assessment must be considered and discussed with patients prior to proceeding with such testing [2]. In the case presented, it was felt that proceeding directly with resection would be the next appropriate step. In schwannoma, the immunohistochemical testing of biopsied or excised specimens, as in the presented case, classically stains positive for the S100 protein (Schwann cells). Additionally, negative staining for specific protein markers can differentiate schwannomas from the more common lesions, notably CD117 (c-kit) and DOG1 in GIST, and desmin and SMA in smooth muscle tumors. Although the presence of S100 occurs in predominately all of the cases, it is not specific for schwannomas. Other tumors that can stain positive for this marker include various carcinomas, melanomas, gliomas, and Langerhans’ cell lesions [12]. Thus, it is the constellation of anatomic, histologic, and immunologic stains, collectively, that makes a diagnosis.

Complete resection, usually with a wedge gastric resection, is considered a definitive and curative treatment [2]. Minimally invasive laparoscopic or robotic techniques provide an opportunity for complete resections and expedited recovery, less morbidity, and therefore appear to be the option of choice [2]. In the presented octogenarian, laparoscopic resection allowed for a quick and smooth postoperative course with a short hospital stay. More recently, endoscopic resection, a technique first successfully performed in 2013, has become an option to consider in tumors <3 cm in diameter. Larger masses associated with deeper locations or layers, unclear boundaries, or metastasis are considered contraindications to this approach [2]. The endoscopic procedure typically involves the removal of the mucosa or a full-thickness resection. Endoscopic resection carries the risk of bleeding, gastrointestinal perforation, and fistula formation, but has not been associated with an increased risk of recurrence or metastasis [13]. 

Schwannomas generally run a benign course. While gastric schwannomas are already rare, reports of metastasis are even rarer, reported in only 2%, and are typically seen in lymph nodes [11]. Patients with metastasis tend to have a poor prognosis with a 30% risk of mortality related to metastasis or recurrence of the tumor within 5 years of surgery [13]. Features associated with the risk of recurrence or metastasis include metastasis to lymph nodes at presentation, tumor size ≥5 cm, lymphovascular invasion, mitotic figures greater than 10/50 high power fields, and incomplete surgical resection [13]. In the presented case, given the tumor size, excision with negative margins, lack of lymph node involvement, and a low Ki-67, the patient was considered cured, with no need for follow-up, similar to other reported cases [11]. It is to be noted that the spreading of a schwannoma is distinct from GISTs, with GISTs having a much higher rate of spread, almost tenfold higher, and mostly spreading hematogenously with liver metastasis. Accordingly, the five-year survival in cases of metastatic GIST is lower and reported to be at 52% from surgical treatment and/or systemic therapy [14].

In the presented case, the non-tumor gastric tissue was found to have chronic gastritis with a *H. pylori *infection. It is reported that *H. pylori *increases the risk of gastric adenocarcinomas and gastric MALT lymphomas [15]. However, such correlations with schwannoma have not been studied and remain unknown. Genetic studies show that the NF2 gene on chromosome 22 plays an essential role in sporadic and syndromic schwannoma development. The NF2 gene encodes for the merlin protein (schwannomin). Specific mutations in the NF2 gene cause the inactivation of the gene, thus preventing the formation of the merlin protein. Inactivation of both alleles of the NF2 gene is observed in most schwannomas. The Carney complex may have a loss of *PRKAR1A* (Protein Kinase CAMP-Dependent Type I Regulatory Subunit Alpha) expression [16].

## Conclusions

In conclusion, while an isolated gastric schwannoma is a rare diagnosis with a nonspecific clinical presentation, it is important to include it in the differential diagnosis when an intramural gastric mass is identified. Definitive diagnosis rests on the pathological findings. Complete resection, preferably with minimally invasive techniques, is considered a definitive and curative approach for treatment, and in general, no follow-up is needed. Further research is warranted to better define the associated risk factors, the pathogenesis of this uncommon tumor, as well as worrisome features that might warrant post-resection surveillance. 
